# Microsporidia in Commercially Harvested Marine Fish: A Potential Health Risk for Consumers

**DOI:** 10.3390/ani13162673

**Published:** 2023-08-19

**Authors:** Samantha Moratal, Angela Magnet, Fernando Izquierdo, Carmen del Águila, Jordi López-Ramon, María Auxiliadora Dea-Ayuela

**Affiliations:** 1 Servicio de Análisis, Investigación y Gestión de Animales Silvestres (SAIGAS), Facultad de Veterinaria, Universidad Cardenal Herrera-CEU, CEU Universities, C/Tirant lo Blanc, Alfara del Patriarca, 46115 Valencia, Spainjordi.lopez1@uchceu.es (J.L.-R.); 2 Facultad de Farmacia, Universidad San Pablo-CEU, CEU Universities, Urbanización Montepríncipe, 28660 Boadilla del Monte, Spain; 3 Wildlife Ecology & Health Group (WE&H), Veterinary Faculty, Universitat Autònoma de Barcelona (UAB), Travessera dels Turons, Bellaterra, 08193 Barcelona, Spain; 4 Departamento Farmacia, Facultad de Ciencias de la Salud, Universidad Cardenal Herrera-CEU, CEU Universities, C/Ramón y Cajal, Alfara del Patriarca, 46115 Valencia, Spain

**Keywords:** *Encephalitozoon hellem*, *Encephalitozoon intestinalis*, *Enterocytozoon bieneusi*, real-time PCR, zoonosis

## Abstract

**Simple Summary:**

Microsporidia are widespread fungal pathogens that affect several organisms, including humans, and are transmitted by food or water. This study aims to survey the presence of the main human-pathogenic microsporidia in digestive samples of edible marine fish in the Comunidad Valenciana, Spain. For this purpose, 138 farmed fish and 113 wild fish from commercial fishing were simultaneously tested using a specific polymerase chain reaction assay. We detected, for the first time, the presence of the zoonotic species *Encephalitozoon intestinalis/hellem* in both studied fish groups, together with the presence of microsporidia from the family Enterocytozoonidae. In marine fish, *E. intestinalis/hellem* suggests a potential risk for public health. However, additional studies in the characterization and epidemiology of these pathogenic microsporidia species in fish are necessary.

**Abstract:**

Microsporidia are widely spread obligate intracellular fungal pathogens from vertebrate and invertebrate organisms, mainly transmitted by contaminated food and water. This study aims to detect the presence of major human-pathogenic microsporidia, i.e., *Enterocytozoon bieneusi*, *Encephalitozoon intestinalis*, *Encephalitozoon hellem*, and *Encephalitozoon cuniculi*, in the gastrointestinal tract of commercially harvested marine fish from Mediterranean coast of the Comunidad Valenciana, Eastern Spain. A total of 251 fish, 138 farmed fish and 113 wild fish from commercial fishing were tested by SYBR Green real-time PCR, enabling the simultaneous detection of the four targeted species. *E. intestinalis/hellem* was found in 1.45% of farmed fish and 7.96% of wild fish, while Enterocytozoonidae was detected in 2.90% and 18.58% of farmed and wild fish, respectively. *E. cuniculi* was not detected in any of the analyzed specimens. To the authors’ knowledge, this is the first report of *E. intestinalis/hellem* in fish, particularly in marine fish. Although the role of fish in these species’ epidemiology remains unknown, this finding points out a potential public health risk linked to fish consumption. Further studies are necessary to characterize these microsporidia in fish hosts better and to elucidate their epidemiological role.

## 1. Introduction

Microsporidia is a wide and diverse phylum that comprises more than 1500 species of obligate intracellular and spore-forming parasites, currently classified as fungi [1]. The general life cycle comprises three phases. During the infective or environmental phase, the spores are liberated and dispersed in the environment, being environmental conditions a key factor for its germination; spores constitute the free-living, resistant, and infective form. Hosts could become infected by ingestion or inhalation of the spores. Once in the host, the spores infect the cells actively by either injecting their sporoplasm through a unique structure, the extrusion apparatus, or passively by being phagocytized [2]. Recent studies suggest a third via of infection by the formation of a synapsis pocket at the surface of the host cell mediated by the polar tube proteins, where the sporoplasm is deposited and lately engulfed inside the host cell [3]. Independently of how microsporidia infect the host cell, the proliferative and sporogonic phases occur inside the cell. Depending on the species, this could occur in the cytoplasm of the host-infected cell, inside a parasitophorous vacuole, or inside the nucleus [2].

Microsporidia infects an extensive host range, comprising both vertebrates, including humans, and invertebrate hosts. Almost half of the 220 microsporidia genera described are known to infect aquatic organisms [4], with about 20 genera infecting fish [5]. According to the microsporidia species and tissue affected, different clinical and pathological signs can be observed in fish, like emaciation, growth inhibition, or leukemia-like syndrome. Moreover, some genera are known to induce xenome formation (*Glugea* spp. or *Loma* spp.) with high-tissue destruction [6]. Consequently, fish microsporidia impact the production of farmed populations with significant economic losses in the aquaculture sector. Their ability to infect different tissues and organs leads to reduced growth rates, decreased feed conversion efficiency, and high mortality rates. For example, *Tetramicra brevifilum* was associated with an outbreak of high mortalities in cultured turbot (*Scopthalmus maximus*) from Galicia (Spain) [7]. More recently, *Enterospora nucleophila* was identified as being responsible for emaciative syndrome and mortalities in gilthead seabream (*Sparus aurata*) cultures from Mediterranean waters [8]. Microsporidian infections can also impact wild fish populations and have been associated with the collapse of some fisheries, therefore affecting commercial fishing [9].

However, little is known about the presence of zoonotic microsporidia in fishes. Currently, nine genera are recognized to cause infection in humans, namely *Anncaliia*, *Encephalitozoon*, *Enterocytozoon*, *Microsporidium*, *Nosema*, *Pleistophora*, *Trachipleistophora*, *Tubulinosema*, and *Vittaforma* [10]. To our knowledge, only the genus *Pleistophora* from poikilothermic hosts (fish and reptiles) has been related to myositis in immunocompromised people; although later analysis classified it in a new species, *Pleistophora ronneafiei*, specific from humans [11]. Within these genera, 17 species are known to infect humans [12]. From those, *Enterocytozoon bieneusi* is the most frequently diagnosed human species, followed by *Encephalitozoon intestinalis*, *Encephalitozoon hellem*, and *Encephalitozoon cuniculi* [13]. Microsporidiosis in humans is mainly associated with a diarrheal syndrome and other affections like encephalitis, keratoconjunctivitis, hepatitis, sinusitis, myositis, or disseminated infection, mainly in immunocompromised people [12,14]. Although, in recent years, they are increasingly recognized in immunocompetent populations including travelers, children, and old age people [15,16,17,18].

Infective spores are very adaptable and resistant to different environmental conditions of temperature, humidity, desiccation and even to water treatments (e.g., water chlorination), facilitating its persistence in the environment and, therefore, its transmission. They are mainly transmitted by the ingestion of contaminated food or water, being the water crucial in the epidemiology of microsporidia species [12,14]. Human pathogenic microsporidia have been found in different water sources like wastewater, drinking water, or recreational river waters [19,20,21,22]. Moreover, microsporidia zoonotic species can also reach the marine environment. For example, *E. bieneusi* has been found in recreational beach water from Chesapeake Bay, Maryland [23], and *E. bieneusi* with *E. cuniculi* have been detected in biofilms from Pensacola Bay, Florida [24]. Like other waterborne pathogens, zoonotic microsporidia are also detected in shellfish in rivers and coastal waters [25,26]. Their presence on shellfish is interesting for biomonitoring purposes but also highlights a potential health risk from aquatic food consumption.

Due to their presence in coastal waters and shellfish, we hypothesize that these major zoonotic microsporidia species could also be present in edible marine fishes, either by parasitizing them or as vectors facilitating their dissemination and posing a potential risk for fish consumers; as has been demonstrated for other zoonotic parasites like *Cryptosporidium* spp., *Toxoplasma gondii*, *Giardia duodenalis* or *Blastocystis* sp. [27,28,29,30]. Therefore, this study aims to assess the potential presence of the main microsporidia zoonotic species (namely *E. bieneusi*, *E. intestinalis*, *E. hellem*, and *E. cuniculi*) in both farmed fish and wild fish from commercial fisheries in the Comunidad Valenciana (Spain), an important aquaculture and fishing area of the Western Mediterranean.

## 2. Materials and Methods

### 2.1. Study Area and Fish Sampling

For the present study, commercial edible fishes from the marine area of the Comunidad Valenciana (Spain), Western Mediterranean (FAO division 37.1.1), were analyzed. A total of 251 fish were sampled, including two differentiated groups: 138 farmed fish and 113 wild fish acquired at fish markets.

Concerning farmed fish, three commercially important species were sampled between July 2020 and October 2021 from four different offshore fattening farms: European sea bass (*Dicentrarchus labrax*), gilthead seabream (*Sparus aurata*), and meagre (*Argyrosomus regius*) (Table S1). The Comunidad Valenciana is the leading producer in Spain for these species, among the top ten species produced by aquaculture in the European Union [31]. This region boasts favorable environmental conditions for the growth and development of these aquaculture species, including suitable water temperatures (ranging from 12 °C in winter to 28 °C in summer), and fair currents ensuring adequate water quality. In this type of production, located near the coastal line, fish are reared in floating pens with nets that allow water circulation and imply direct communication with the surrounding environment.

On the other side, wild fish were donated by fishers from different fish markets of the Comunidad Valenciana between March and June 2021 (Table S2). In this case, the fish came mainly from bottom trawling and were randomly chosen from different ships and species to avoid economic losses to fishermen.

Farmed fish were obtained during regular working operations at participating farms and were stunned and sacrificed by the farm staff following the standard approved methods used in Mediterranean marine aquaculture, consisting of immersion into a slurry ice solution. Wild fish were sampled at fish markets once dead due to commercial fishing activity. Therefore, no ethical approval was required from the Ethics Committee of Animal Experimentation.

Sampled fish were maintained in refrigeration for transportation and storage up to processing within 24 h after death. For each individual, species, body weight, and total body length were recorded, and two types of samples were obtained using sterile dissection material: (1) gastrointestinal tissue scrapings mixed with intestinal content, and (2) somatic muscle from the caudal region. Both samples were frozen without preservatives at −20 °C until further processing.

### 2.2. DNA Extraction

DNA from gastrointestinal tract samples was extracted using the NZY Tissue gDNA Isolation Kit (Nzytech genes & enzymes, Lisbon, Portugal). Manufacturer’s indications were applied: pretreatment steps for stool samples, followed by the standard extraction protocol.

DNA extraction from somatic muscle was performed using the DNeasy Blood and Tissue Kit (Qiagen, Hilden, Germany). The manufacturer’s protocol was followed, considering that samples were incubated with the proteinase k overnight (16–17 h).

### 2.3. Real-Time PCR for Human Pathogenic Microsporidia Detection and Species Identification

Human pathogenic microsporidia species detection and identification was conducted through an SYBR Green real-time PCR, described by Polley et al. [32] and adapted by Andreu-Ballester et al. [33], targeting a region of the small subunit rRNA (SSU rRNA). Briefly, QuantiTect SYBR Green PCR kit (Qiagen, Hilden, Germany) was used in a total reaction volume of 20 µL containing 5 µL of template DNA. MsRTf1 and MsRTr1 primers were added at 0.5 µM final concentrations. Amplification was performed in an Mx3000 qPCR System (Agilent, Santa Clara, CA, USA) with the following cycling conditions: 50 °C for 2 min, 95 °C for 15 min, and 40 cycles of 95 °C for 10 s, 60 °C for 20 s and 72 °C for 20 s. The dissociation curve begins with a 1 min incubation at 95 °C to melt the DNA and then a 30-s incubation at 55 °C, followed by a ramp up to 95 °C with Allpoints data collection performed during the ramp. Data collection during the ramp slows the ramp rate to 0.01 °C/s.

This PCR allows for identifying the most common zoonotic microsporidia species infecting humans according to specific melting temperatures (Tm). *E. cuniculi* amplifies at 84.45 ± 0.4 °C, *E. intestinalis/hellem* at 82.85–83.9 °C (this PCR assay does not permit to distinguish between both species), and finally, *E. bieneusi* at 82.35 ± 0.4 °C. This PCR test was designed for human diagnosis; therefore, the possible cross-reaction with other microsporidia marine species was not studied. This is particularly relevant in the case of *E. bieneusi* since all other species of this family (Enterocytozoonidae) are typically fish or shellfish parasites. That is why the results are presented as either the absence or presence of Enterocytozoonidae. By contrast, *E. intestinalis/hellem* pertains to a family, even extendable to the whole clade, which is eminently terrestrial, with only one marine species parasite of a free-living nematode [34], thus eliminating the possibility of cross-reactions.

Additionally, samples that amplify at a Tm between 80.00 °C and 81.95 °C potentially indicate the presence of other microsporidia species different from those targeted by the real-time PCR employed in this study. Samples with a cycle threshold (Ct) below 30 were subjected to amplicon purification (NucleoSpin^®^ Gel and PCR Clean-up, Macherey-Nagel, Düren, Germany) and subsequent Sanger-sequencing by Macrogen laboratories sequencing service (Korea). Obtained sequences, which showed good quality for further processing, were blasted against microsporidia sequences from the NCBI GenBank database (http://blast.ncbi.nlm.nih.gov/blast, accessed on 16 January 2023). Phylogenetic analysis were conducted using MEGA software, version 11 [35]. The obtained partial sequences were aligned with reference sequences from microsporidia species belonging to the same families/group as reported sequences. The phylogenetic trees were inferred by the Maximum Likelihood (ML) method using the K2 + G substitution model (model selection according to corrected Akaike and Bayesian Information Criteria, AICc and BIC).

## 3. Results

### 3.1. Fish Sampled

Three species of farmed fish were sampled from four offshore fattening farms. From the overall 138 farmed specimens, 57% (N = 79) were European sea bass, with an average weight and length (mean ± standard deviation) of 384.35 ± 189.79 g and 31.16 ± 5.52 cm; 26% (N = 36) were gilthead seabream, with an average weight and length of 278.42 ± 75.95 g and 24.44 ± 2.16 cm; and 17% (N = 23) were meagre, with an average weight and length of 491, 22 ± 276.06 g (the weigh was available only in 12 specimens) and 42.48 ± 11.81 cm.

On the other hand, wild fish acquired at fish markets (N = 113), was a heterogeneous group composed of 31 different species, although 47.79% was represented by only four species. Atlantic mackerel (*Scomber scombrus*) (N = 22; 19.47%) with an average weight and length of 82.90 ± 45.72 g and 21.31 ± 2.66 cm; pouting (*Trisopterus luscus*) (N = 12; 10.62%) with an average weight and length of 51.76 ± 20.53 g and 16.60 ± 2.01 cm; the European hake (*Merluccius merluccius*) (N = 11; 9.73%) with an average weight and length of 67.85 ± 19.77 g and 20.98 ± 1.70 cm; and the red mullet (*Mullus barbatus*) (N = 9; 7.96%) with an average weight and length of 82.18 ± 40.06 g and 19.03 ± 2.42 cm. Fifteen species correspond to unique specimens. Information on all species sampled can be found in Supplementary Table S2.

### 3.2. Zoonotic Microsporidia in Gastrointestinal Tract Samples

Zoonotic microsporidia were detected in 36 out of 251 analyzed fishes (14.34%). Of these, 30 fishes were wild fish from fish markets (26.55%), and only six individuals were farmed fish (4.35%). The most prevalent was Enterocytozoonidae (9.96%), followed by *E. intestinalis/hellem* (4.38%); no co-infections were detected between both in any of the analyzed specimens. All samples were negative for *E. cuniculi* (Table 1). Positive isolates from farmed-raised fish were detected in European sea bass and gilthead seabream from only two of the four sampled farms (Table S1). Concerning wild fish, positives were detected in several species from the different fish markets. *E. intestinalis/hellem* was detected in annular seabream (*Diplodus annularis*), red mullet (*Mullus barbatus*), surmullet (*Mullus surmuletus*), axillary seabream (*Pagellus acarne*), common pandora (*Pagellus erytrinus*), brown comber (*Serranus hepatus*) and Mediterranean horse mackerel (*Trachurus mediterraneus)*, while Enterocytozoonidae was found in annular seabream, blackbelly rosefish (*Helicolenus dactylopterus*), large-scaled gurnard (*Lepidotrigla cavillone*), European hake (*Merluccius merluccius*), blue whiting (*Micromessistius poutassou*), surmullet, greater forkbeard (*Phycis blennoides*), Atlantic mackerel (*Scomber scombrus*), comber (*Serranus cabrilla*) and Mediterranean horse mackerel (Table S2).

### 3.3. Zoonotic Microsporidia in Muscle Samples

Muscle from fishes that were positive at the gastrointestinal tract for Enterocytozoonidae or *E. intestinalis/hellem* was also analyzed to determine if these zoonotic species could reach the edible muscular tissue. None of the 26 analyzed samples were positive. Unfortunately, the remaining 10 specimens could not be analyzed, as muscle samples were not available.

### 3.4. Other Microsporidia Species

**Figure 1:**
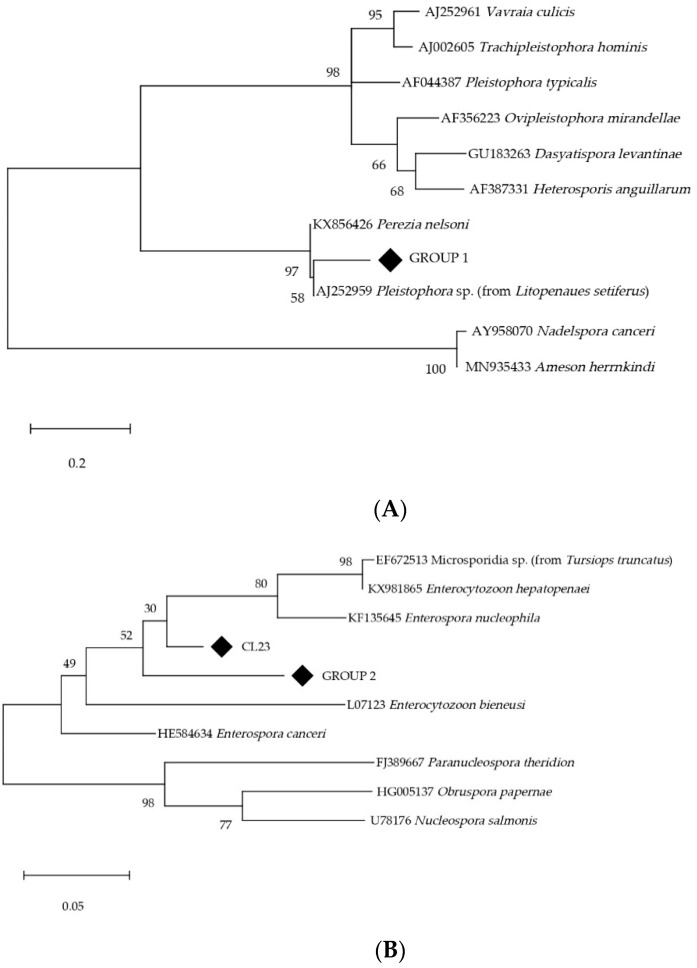
Phylogenetic trees of the other microsporidia reported in this study (◆) were inferred by the Maximum-Likelihood method based on K2 + G substitution model applied to partial SSU rRNA gene sequences. (**A**) Group 1 clustering in family Pereziidae, Glugeida group (273 bp); (**B**) Group 2 and sample CL23 clustering in family Enterocytozoonidae (198 bp). Percentage support from 2000 replicates (bootstrap test) is indicated.

Additionally, 61 out of 251 samples amplified at a Tm between 80.00 °C and 81.95 °C indicated the presence of other microsporidia species different from those targeted. Among these samples, only 18 showed a Ct below 30 and were subjected to Sanger-sequencing. Twelve sequences of approximately 250 bp of the SSU rRNA were obtained, which showed good quality for further processing. Sequences were blasted against microsporidia sequences from the NCBI GenBank database and phylogenetic trees were constructed using MEGA software, version 11 [35], and reference sequences from microsporidia species belonging to the same families/group as reported sequences (Figure 1). The analysis revealed two groups of homologous sequences and one different sequence. Group 1 (N = 6) exhibited ≈93% identity with one isolate from the decapod *Litopenaeus setiferus* (AJ252959). Although the authors found the closest similarity with *Pleistophora* sp., our phylogenetic reconstruction showed that it grouped with Pereziidae, a family of microsporidia infecting marine decapods, which clusters in the Glugeida group together with Pleistophoridae [36] (Figure 1A). Group 2 (N = 5) exhibited 91–92% identity with a non-characterized microsporidian isolated (EF672513) from the common bottlenose dolphin (*Tursiops truncatus*), clustering within the family Enterocytozoonidae [34] (Figure 1B). Finally, the latest sequence (CL23) showed 93.31% homology with *E. nucleophila* (KF135645), a member of the Enterocytozoonidae family (Figure 1B). Obtained partial sequences of the SSU rRNA gene have been deposited in GenBank under the following accession numbers: OR001667-OR001678.

## 4. Discussion

Here, we detect the presence of zoonotic microsporidia species *E. intestinalis/hellem* in marine fish for the first time. This new epidemiological information is of high interest, especially if we take into account that microsporidia are currently recognized as emerging pathogens, listed by The National Institute of Allergies and Infectious Diseases (NIAID) as emerging infectious pathogens (Category B) of high priority for public health risk (https://www.niaid.nih.gov/research/emerging-infectious-diseases-pathogens, accessed on 10 May 2023). It is also well-known that water plays an essential role in microsporidian spores’ survival and transmission to humans, either for direct consumption, food irrigation, or recreational bathing [37,38]. Therefore, fish could obtain spores from marine water contaminated by infected humans and animals.

To identify the microsporidia species in the fish samples, we utilized an SYBR green real-time PCR, as previously described by Polley et al. [32], which allows the simultaneous detection and species identification of the main human pathogenic microsporidia species. These assays are appropriate when the expected parasite load is low, as is foreseeable in the case of fish acting as passive carriers. They offer better sensitivity compared to conventional PCR techniques and simultaneously enable multiple species testing, facilitating a comprehensive analysis even with limited DNA samples. However, the limitation of this method is that it does not allow for genotyping of the isolates.

Complementary morphological identification could not be performed due to the sample conservation method. Samples in this study were part of a broader project and, therefore, they were directly preserved in frozen to avoid any DNA degradation. Morphological identification based on staining techniques can be used as a complementary diagnostic method to identify the presence of microsporidia, but cannot be used to differentiate between species. Conversely, the use of transmission electron microscopy, although less practical and expensive, can be used as a reliable technique for species morphological diagnosis based on the spore size, the number of polar tube coils, and the presence/absence and characteristics of the parasitophorous vacuole [2,39].

The highest presence (9.96%) was detected for Enterocytozoonidae, potentially *E. bieneusi*, which is the most common microsporidian species diagnosed in humans [40], but also present in a wide range of birds and mammals [41,42]. Interestingly, this species groups in a family of microsporidians whose other components infect only aquatic hosts (fish and crustaceans) [5,34]. *E. bieneusi* was also detected in coastal waters [23,24] and filter-feeding mollusks (e.g., [26]). Nevertheless, the question remains about their role: authentic hosts or passive carriers. Similarly, we are reporting the first evidence of the potential presence of *E. bieneusi* in fish hosts, although we also cannot discern whether an infection is occurring or, if on the contrary, fish obtained the parasite in their digestive tracts from the water and/or by the feeding on aquatic invertebrates. Regardless, the close phylogenetic relationship between *E. bieneusi* and other aquatic microsporidians enhances the interest in elucidating the epidemiological role that water and aquatic hosts could play in *E. bieneusi* human infections. In this regard, it is essential to consider the enormous genetic variability exhibited by *E. bieneusi*. Based on the analysis of the ribosomal internal transcribed spacer (*ITS*) region, several genotypes of *E. bieneusi*, distributed in 11 groups and with different host specificities, have been described. Zoonotic genotypes from group 1 are among the most frequently reported in different water sources [43]. Further studies delving deeper into *E. bieneusi* genotyping in aquatic hosts should be addressed to ascertain the role that seafood and, in particular, fish, might play in *E. bieneusi* epidemiology and public health.

A presence of 4.38% was detected for zoonotic *E.intestinalis/hellem*. *E. intestinalis* is the second most common cause of microsporidian diarrhea in humans, only behind *E. bieneusi* [14], and, interestingly, it has been identified in recreational water rivers from northern Spain [21]. *E. hellem* causes disseminated infections and has not been associated with diarrheal syndrome. It is commonly associated with keratoconjunctivitis and also affects respiratory and urinary systems [44]. Conversely to Enterocytozoonidae, both these species pertain to a family whose members are primarily terrestrial [36], suggesting that, in this case, fish are most probably acting as passive carriers.

The other *Encephalitozoon* species tracked in this survey, namely *E. cuniculi*, was not detected in any of the sampled fishes, although it is also present in water environments [22,24]. *E. cuniculi* is the fourth most common microsporidian species found in humans. It can occasionally cause intestinal disease, but it is most commonly found affecting the brain and kidneys [44].

Although we retrieved other microsporidia sequences from this study, their phylogenetic classification must be interpreted cautiously, as it relies on short fragments of the SSU rRNA gene. Nonetheless, the information provided is in congruence with the samples’ nature, encompassing these sequences into two families of marine microsporidia, i.e., Pereziidae and Enterocytozoonidae.

Over the last six decades, the global average consumption per capita of seafood has doubled, influenced by greater supply, changes in dietary habits, nutrition awareness, and an increase in incomes, although, with wide variation between regions and countries. From the total per capita intake, 75% comes from finfish, the rest being shellfish. In turn, marine finfish accounted for the 33% [45]. This study focuses on farmed-raised and wild fish from commercial fisheries, the primary marine fish sources that contribute to supplying this high and still increasing demand for marine fish intended for human consumption [45]. It is worth mentioning that a higher presence was detected in wild fishes (26.55%) compared to the low presence in the farmed group (4.35%). Open-net pens limit farmed fish space, which prevents them from foraging in their natural habitat. Hence, their nutrition relies almost exclusively on provided extruded feed, except for the eventual ingestion of small crustaceans and mollusks that can go through the net [46]. On the other hand, wild fish are unrestricted in their movements and obtain food from the natural environment. Therefore, wild fish commonly feed on shellfish, which can serve as reservoirs and carriers for these zoonotic microsporidia [25,26]. Additionally, wild fish are more likely to come into contact with polluted areas where various contaminants, including pathogens like microsporidia, may be present (e.g., sewage discharges; [47]).

Notably, most of the fish specimens used in this study were originally collected as part of a broader investigation aimed at identifying *Cryptosporidium* spp. [48]. Curiously, the presence in the farmed group was similar (4.8%) to that found for Microsporidia (4.35%). Nevertheless, the presence in wild fish from extractive fisheries was very different for both pathogens, *Cryptosporidium* and Microsporidia (0.9% vs. 26.55%).

The absence of Enterocytozoonidae and *E. intestinalis/hellem* in the muscular tissue of positive fish is under the hypothesis that fish act merely as passive carriers. Even if they were natural hosts, these species commonly affect the gastrointestinal tract and other inner organs [44]. Despite this, there is still a potential risk for the consumer due to cross-contamination during handling and evisceration [20,49]. This is especially relevant considering that the consumption of raw and undercooked fish dishes has notably increased in recent years in Western countries [50]. Finally, to better characterize the risk of acquiring microsporidiosis from fish consumption, it would be necessary to perform studies addressed to assess the infectivity of the spores contained in the gastrointestinal tract of fish.

## 5. Conclusions

This work is, to our knowledge, the first survey conducted on the presence of human pathogenic microsporidia in commercial edible marine fish, both farmed fish and wild fish from extractive fisheries.

In conclusion, this study provides valuable insights into the presence of zoonotic microsporidia species, reporting for the first time the presence of *E. intestinalis/hellem*, as well as a high presence of Enterocytozoonidae (potentially *E. bieneusi*) in marine fish. It highlights the potential role of water and aquatic hosts in the epidemiology of these microsporidia and emphasizes the need for further research to understand their impact on public health. Additionally, the contrasting presence rates between farmed and wild fish underscore the importance of considering the source and environmental factors when assessing the risk of microsporidia transmission to humans through fish consumption.

## Figures and Tables

**Table 1:** Presence of zoonotic microsporidia in gastrointestinal tracts from farmed and wild marine fishes in the Comunidad Valenciana, Western Mediterranean.

Group	Presence (Number of Positives/Total)			
	Enterocytozoonidae	*E. intestinalis/hellem*	*E. cuniculi*	Total
**Farmed fish**	2.90% (4/138)	1.45% (2/138)	0% (0/138)	**4.35% (6/138)**
**Wild fish**	18.58% (21/113)	7.96% (9/113)	0% (0/113)	**26.55% (30/113)**
**Total**	**9.96% (25/251)**	**4.38% (11/251)**	**0% (0/251)**	**14.34% (36/251)**

## Data Availability

The data presented in this study are available on request from the corresponding authors.

## Supplementary Materials

The following supporting information can be downloaded at: https://www.mdpi.com/article/10.3390/ani13162673/s1. Table S1: Farmed species sampled. Sampling data, location, and number of positives at the gastrointestinal tract are indicated; Table S2: Wild species sampled. Sampling data, location, and number of positives at the gastrointestinal tract are indicated.
