# Supplementary material for: Microsporidia in Commercially Harvested Marine Fish: A Potential Health Risk for Consumers
Source: Animals (Basel). 2023 Aug 19;13(16):2673. doi: 10.3390/ani13162673 (PMC10451485; doi:10.3390/ani13162673)
Supplement: Supplementary file 1 [file animals-13-02673-s001.zip › animals-2437632-supplementary.pdf]

Table S1. Farmed species sampled. Sampling data, location, and number of positives at the gastrointestinal tract are indicated.

Species	Sampling data	Location	Number of individuals	Enterocytozoonidae positives	*E. hellem*/*E. intestinalis* positives
**European sea bass (*Dicentrarchus labrax*)**	14/07/2020	Farm 4	12	2	1
	24/07/2020	Farm 2	14	1	1
	15/09/2020	Farm 1	14	0	0
	21/10/2020	Farm 2	10	0	0
	05/05/2021	Farm 1	10	0	0
	12/05/2021	Farm 3	9	0	0
	21/07/2021	Farm 3	10	0	0
			**79**	**3**	**2**
**Gilthead seabream (*Sparus aurata*)**	09/09/2020	Farm 4	14	0	0
	15/10/2020	Farm 4	14	1	0
	07/10/2021	Farm 1	8	0	0
			**36**	**1**	**0**
**Meagre (*Argyrosomus regius*)**	29/09/2020	Farm 2	12	0	0
	07/10/2020	Farm 3	11	0	0
			**23**	**0**	**0**
**Total cultivated fish**			**138**	**4**	**2**

Table S2. Wild species sampled. Sampling data, location, and number of positives at the gastrointestinal tract are indicated.

Species	Sampling data	Location	Number of individuals	Enterocytozoonidae positives	*E. hellem*/*E. intestinalis* positives
**Argentine (*Argentina sphyraena*)**	02/06/2021	Fish market 4	1	0	0
**Meagre (*Argyrosomus regius*)**	02/06/2021	Fish market 4	1	0	0
**Imperial scaldfish (*Arnoglossus imperialis*)**	02/06/2021	Fish market 4	1	0	0
**Bogue (*Boops boops*)**	15/03/2021	Fish market 2	3	0	0
**Red gurnard (*Chelidonichthys cuculus*)**	29/06/2021	Fish market 3	1	0	0
**Spotted flounder (*Citharus linguatula*)**	30/03/2021	Fish market 3	1	0	0
	02/06/2021	Fish market 4	1	0	0
	29/06/2021	Fish market 3	3	0	0
**European conger (*Conger conger*)**	09/03/2021	Fish market 4	2	0	0
**Annular seabream (*Diplodus annularis*)**	15/03/2021	Fish market 2	2	1	1
**Common two-banded seabream (*Diplodus vulgaris*)**	02/06/2021	Fish market 4	1	0	0
**Blackbelly rosefish (*Helicolenus dactylopterus*)**	02/06/2021	Fish market 4	1	1	0
**Brown wrasse (*Labrus merula*)**	02/06/2021	Fish market 4	1	0	0
**Large-scaled gurnard (*Lepidotrigla cavillone*)**	02/06/2021	Fish market 4	1	1	0
**Blackbellied angler (*Lophius budegassa*)**	02/06/2021	Fish market 4	1	0	0
	29/06/2021	Fish market 3	1	0	0
**European hake (*Merluccius merluccius*)**	09/03/2021	Fish market 4	3	0	0
	15/03/2021	Fish market 2	2	2	0
	02/06/2021	Fish market 4	1	0	0
	29/06/2021	Fish market 3	5	1	0
**Blue whiting (*Micromessistius poutassou*)**	09/03/2021	Fish market 4	2	0	0
	30/03/2021	Fish market 3	2	1	0

	02/06/2021	Fish market 4	1	1	0
**Red mullet (*Mullus barbatus*)**	09/03/2021	Fish market 4	1	0	0
	15/03/2021	Fish market 2	1	0	1
	30/03/2021	Fish market 3	3	0	0
	02/06/2021	Fish market 4	1	0	0
	29/06/2021	Fish market 3	3	0	0
**Surmullet (*Mullus surmuletus*)**	09/03/2021	Fish market 4	2	0	1
	30/03/2021	Fish market 3	1	1	0
**Axillary seabream (*Pagellus acarne*)**	15/03/2021	Fish market 2	1	0	1
**Common pandora (*Pagellus erytrinus*)**	15/03/2021	Fish market 2	4	0	1
	30/03/2021	Fish market 3	1	0	0
	02/06/2021	Fish market 4	1	0	1
**African armoured searobin (*Peristedion cataphractum*)**	09/03/2021	Fish market 4	1	0	0
**Greater forkbeard (*Phycis blennoides*)**	09/03/2021	Fish market 4	3	0	0
	30/03/2021	Fish market 3	4	0	0
	02/06/2021	Fish market 4	1	1	0
**Forkbeard (*Phycis phycis*)**	02/06/2021	Fish market 4	1	0	0
**Salema (*Sarpa salpa*)**	02/06/2021	Fish market 4	1	0	0
**Atlantic mackerel (*Scomber scombrus*)**	09/03/2021	Fish market 4	2	0	0
	15/03/2021	Fish market 2	1	0	0
	14/06/2021	Fish market 1	19	8	0
**Lesser spotted dogfish (*Scyliorhinus canicula*)**	09/03/2021	Fish market 3	3	0	0
**Comber (*Serranus cabrilla*)**	09/03/2021	Fish market 4	1	1	0
**Brown comber (*Serranus hepatus*)**	09/03/2021	Fish market 4	1	0	1
	30/30/2021	Fish market 3	1	0	0
**Greater weever (*Trachinus draco*)**	30/03/2021	Fish market 3	1	0	0
**Mediterranean horse mackerel (*Trachurus mediterraneus*)**	15/03/2021	Fish market 2	2	1	0
	02/06/2021	Fish market 4	1	0	1
**Pouting (*Trisopterus luscus*)**	09/03/2021	Fish market 4	1	0	0
	15/03/2021	Fish market 2	2	0	0
	30/03/2021	Fish market 3	6	0	0
	02/06/2021	Fish market 4	1	0	0
	29/06/2021	Fish market 3	2	0	0
**Stargazer (*Uranoscopus scaber*)**	02/06/2021	Fish market 4	1	0	0
**Total fish from fish markets**			**113**	**20**	8
